# Introducing a new index for selecting genetic polymorphisms for association studies

**DOI:** 10.17179/excli2022-5004

**Published:** 2022-06-10

**Authors:** Nafiseh Omidpanah, Mostafa Saadat

**Affiliations:** 1 Department of Biology, College of Sciences, Shiraz University, Shiraz, Iran

## ⁯

As a result of the introduction and development of next-generation sequencing technologies, enormous genetic alterations have been detected in the human genome. Some of these variations are disease-causing variations of Mendelian disorders and many single nucleotide genetic polymorphisms (SNPs) are associated with multifactorial complex traits such as cancers. Distinguishing which of these massive genetic variants are causes of Mendelian inheritance diseases and which are 'influential' to multifactorial traits is of great importance and very challenging for research activity.

Numerous computational methods (e.g., PolyPhen, SIFT, and GERP) have been developed for ranking effect(s) of single nucleotide variants (SNVs) and short insertion/deletions on human genomes in the post-genome era. Combined Annotation-Dependent Depletion (CADD) is one of these computational methods. It is an integrative index built on the basis of more than 60 genomic features (including GERP, ENCODE, phyloP, SIFT, PolyPhen) to measure the risk of genetic variations in the human genome. Therefore, this method delivers a more reliable evaluation of SNVs deleteriousness. CADD tool scores (C-scores) are calculated on several genomic features derived from evolutionary constraints, surrounding sequence context, epigenetic measurements, functional predictions, and gene model annotations (Kircher et al., 2014[4]; Rentzsch et al., 2019[7]). CADD is a widely used index, measuring the causal effects of SNVs' in severe human Mendelian disorders.

In a recent study, the predicted risk of genetic variants data in the ClinVar and VariBench databases, which was estimated by CADD, was compared with those predicted by 13 other important computational methods. This study showed that the area under the Receiver Operating Characteristic (ROC) curve of CADD was the highest value compared to the other methods, indicating excellent performance of CADD (Wang et al., 2022[10]). Higher C-scores are more likely to be deleterious. The C-scores below and above 30 are assigned as 'likely benign' and 'likely deleterious', respectively. Variants with C-scores over 30 are predicted to be about 0.1 % in the human genome (Rentzsch et al., 2019[7]).

In Mendelian inheritance diseases, each allele has a very important effect on the pathogenesis of a disease. Therefore, genetic variants with very high C-scores are good targets to find the mutations involved in the development of autosomal and dominant diseases (Thiha et al., 2019[9]; LeDoux, 2020[5]; Shinwari et al., 2021[8]; Jamiri et al., 2022[3]). In contrast, in multifactorial diseases, numerous genetic and environmental elements are involved in the development of diseases and each gene and its alleles have small effects on the pathogenesis of the disease. In a study of more than 3000 genetic variants with MAF <0.01 and C-score >20, only 27 variants were identified as high-risk mutations in colorectal cancer. More than 50 % of these mutations were detected in three out of 50 studied families (Helgadottir et al., 2021[2]), indicating that rare SNVs cannot explain the development of colorectal cancer in human populations. In genetic association studies (mainly with case-control design), researchers often test one or a few SNPs of a given gene. Prediction of the functional impact of each SNP is very important for choosing the potential 'influential' SNPs in genetic association studies. The aim of the present report is to assist researchers to select appropriate SNPs in their association studies.

In this study we used SNVs of *XRCC1* (MIM 194360), *XPC* (MIM 613208), *PPP1CA* (MIM 176875), *SOD1* (MIM 147450), and *TERT* (MIM 187270) genes with the global minor allele frequencies (MAFs) equal or higher than 0.0002. The C-scores and MAFs were obtained from e!Ensembl (https://asia.ensembl.org/Homo_sapiens/Info/Index) using Human genome assembly GRCh38.p13 (see Supplementary Table 1).

In total, 4145 SNVs were included in the analysis. From these, 93.3 % of SNVs have C-scores less than 10 and only 8 SNVs have C-scores higher than 30. Present data shows that 88.3 % of SNVs have MAF less than 1 % (Supplementary Figure 1). Supplementary Figure 1 also shows the scatter diagram of C-scores *vs* MAFs. The majority of SNVs had C-scores less than 10 and MAFs less than 1 %. SNVs with a C-score >30 have very low MAFs. Statistical analysis showed that there was a negative significant relationship between C-scores and LnMAFs in a regression analysis without constant term in the equation (r=-0.603, P<0.001). Similar results were obtained when individual studied genes were analyzed (data not shown).

In epidemiological studies, investigators frequently use three terms: relative risk, attributable risk and population attributable risk. “Relative risk” is the ratio of incidence rates of a health condition in two groups, those exposed to a factor and those not exposed. “Attributable risk” shows the excess risk from exposure to a particular risk factor and “population attributable risk” indicates the proportion of the incidence of a disease at the population level which is due to a particular exposure. To compute population attributable risk, the attributable risk is multiplied by the prevalence of exposure in the population (Bonita et al., 2006[1]).

The C-score predicts the deleteriousness of SNVs. This score is strongly correlated with the relative risk and attributable risk of a specific genetic variant. It is well established that in multifactorial traits, each polymorphism has small relative and attributable risks. Therefore, the C-score alone is not a suitable indicator for choosing SNPs for genetic association studies. 

If a given SNP is associated with the risk of a disease which is prevalent in gene pools, it has a high level of population attributable risk. To compute the population attributable risk, the attributable risk is multiplied by the prevalence of exposure in the population. In a similar way, we introduce a combined index which is calculated by the C-score multiplied by the MAF. It is obvious that this index is not population attributable risk for a given genetic polymorphism, but they are highly correlated with each other. We call the introduced index “polymorphism selection index” (PSI). The new introduced index can help researchers to select a few SNPs among the large number of SNPs than the C-score index alone. 

In order to examine the efficiency of the PSI, we focused on common SNPs in the X-ray cross complementing protein 1 gene (*XRCC1*). The XRCC1 is a scaffold protein involved in single strand break repair, base excision repair, and in other repair pathways (London, 2020[6]). The *XRCC1* has three common polymorphisms named Arg194Trp (rs1799782), Arg280His (rs25489), and Arg399Gln (rs25487). It is well established that reduction in cellular DNA repair capacity is involved in the development of various types of malignancies. Therefore, it is suggested that polymorphisms of *XRCC1* might be associated with cancer risk. The latest relevant meta-analyses were found by searching through PubMed. Search keywords have been *XRCC1*, meta-analysis and polymorphism. The following data were extracted from the eligible meta-analysis: type of malignancy, type of polymorphism, and the result of the meta-analysis (see Supplementary Table 2). 

Supplementary Table 3 summarizes the C-scores, MAFs, number of malignancies statistically associated with each polymorphism, number of malignancies not associated with the polymorphisms, and number of malignancies for which no published meta-analysis of polymorphisms is available.

Based on the data presented in Supplementary Table 3, 61.1, 13.3 and 66.7 percent of cancer types were significantly associated with the Arg194Trp, Arg280His, and Arg399Gln polymorphisms, respectively. An interesting observation was the significant linear trend between the number of malignancies which are significantly associated with SNPs and PSIs (χ^2^ for linear trend =10.93, df=1, P=0.001). It should be noted that there was no significant linear trend between the number of malignancies which are significantly associated with SNPs and C-scores (χ^2^ for linear trend =1.71, df=1, P=0.190). In order to rule out the influence of the number of malignancies, which still were not studied with the SNPs, on the above-mentioned trend, the “sensitivity analysis” was performed. We tested two assumptions: assumption I: 70 % of these malignancies were assumed to be associated with SNPs; and alternatively, 70 % of malignancies were assumed to not be associated with SNPs. Under both assumptions, the number of malignancies, which were significantly associated with each SNP, was a function of PSIs (Assumption I: χ^2^ for linear trend =7.08, df=1, P=0.008; Assumption II: χ^2^ for linear trend =10.52, df=1, P=0.001). Taken together, these data support applying the introduced index to select SNPs for genetic association studies.

The present study revealed that the introduced index, which we called “polymorphism selection index” (PSI = C-score × MAF) is a more reliable index than C-score alone for selecting single nucleotide polymorphisms for genetic association studies. A higher PSI indicates that a genetic variation is more likely to be deleterious and suitable for a genetic association study.

## Declaration

### Acknowledgment

The authors would like to thank Dr. Maryam Ansari-Lari and Miss Ellie H. Sharif for their helpful discussion and editing the article.

### Conflict of interest

None. 

## Supplementary Material

Supplementary information
